# Changes of Hepcidin, Ferritin and Iron Levels in Cycling Purebred Spanish Mares

**DOI:** 10.3390/ani13071229

**Published:** 2023-03-31

**Authors:** Katiuska Satué, Esterina Fazio, Deborah La Fauci, Pietro Medica

**Affiliations:** 1 Department of Animal Medicine and Surgery, Faculty of Veterinary Medicine, CEU-Cardenal Herrera University, 46115 Valencia, Spain; 2 Department of Veterinary Sciences, Veterinary Physiology Unit, Messina University, Viale Palatucci 13, 98168 Messina, Italy

**Keywords:** hepcidin, ferritin, iron, ovarian hormones, mares

## Abstract

**Simple Summary:**

The present study provides new insights into the physiological changes in iron metabolism associated with the estrus cycle in Purebred Spanish mares. Relationships between estradiol-17β and progesterone with the levels of iron, ferritin, and hepcidin were evaluated. There is a relationship between progesterone concentrations with levels of iron, ferritin, and the estrous cycle, although with a different trend depending on the type of dominant hormone. Estrogen dominance during estrus appears to exhibit a more efficient iron status under hepcidin inhibition than progesterone during the luteal phase, as both circulating levels and iron stores are reduced due to hepcidin stimulation.

**Abstract:**

Several studies have demonstrated that in woman the sex hormones such as estrogen (E2) and progesterone (P4) influence iron (Fe) regulation, contributing to variations in Fe parameters along the menstrual cycle. These mechanisms based on the regulation of hepcidin (Hepc) which limits Fe availability during the cycle, remain poorly characterized in healthy mares. The objective of this study was to establish the relationship between Hepc, Fe, ferritin (Ferr), and the primary ovarian hormones E2 and P4 in cycling Purebred Spanish mares. Blood samples were taken from 31 Purebred Spanish mares day −5, on day 0, day +5 and day +16 of the cycle. Fe and Ferr significantly increased and Hepc decreased during pre- and ovulatory periods. The secretion peak of estradiol-17β (E2) was reached on day 0 and progesterone (P4) between days +5 and +16. Fe and Ferr were positively correlated (r = 0.57). Fe and Ferr were negatively correlated with Hepc (r = −0.72 and r = −0.02, respectively). E2 and P4 were negatively and positively correlated with Hepc (r = −0.753 and r = 0.54, respectively). In cycling Purebred Spanish mares there is a measurable relationship between steroid hormones and systemic Fe metabolism. Estrogenic dominance in the pre- and ovulatory period allows for a more effective iron status, mediated by hepcidin inhibition. However, P4 during the luteal phase substantially reduces serum Fe and iron stores, possibly related to Hepc stimulation. Future research is required to clarify the relationship between steroid hormones and iron metabolism at the molecular level in equids.

## 1. Introduction

Body iron (Fe) depends primarily on the coordinated interaction between erythropoiesis, the absorption of Fe from the diet by enterocytes, the recycling of Fe by macrophages after degradation from senescent erythrocytes, and the release to plasma from stores in the form of ferritin (Ferr). Most of the Fe in the body is found as part of the haemoglobin contained in erythrocytes. The daily erythrocyte destruction of senescent erythrocytes, which in horses have a half-life of ~140–150 days, is recycled by erythropoiesis [1]. In horses, of all the processes that supply Fe, erythrocyte recycling by macrophages represents approximately 85% of Fe requirements, and the remaining 15% depends on Fe absorption in the small intestine [2]. The Fe availability is controlled by the liver peptide hormone hepcidin (Hepc). The body Fe increase causes the production of hepcidin, which is released in the circulation and acts on its receptor ferroportin (Fpn), a transmembrane Fe exporter protein highly expressed on duodenal enterocyte, macrophages, and hepatocytes [3]. The binding of Hepc to Fpn on the membranes of iron-exporting cells induces the endocytosis and proteolysis of Fpn, and thereby decreases the delivery of Fe to plasma [4] from absorptive duodenal cells and Fe recycling macrophages by blocking its export [5]. Ferric Fe resulting from oxidation of Fe released by Fpn is taken up by transferrin which transports it mainly to the liver for storage and the bone marrow for production of erythrocytes. Hepc hormone regulation occurs through many different stimuli, including iron status, interleukin-6 (IL-6) increases, inflammation, hypoxia, and sex hormones, among others [4]. If Hepc levels decrease, its inhibitory effects diminish, and more Fe is made available from the diet and from the storage pool in macrophages and hepatocytes. On the contrary, overexpression of Hepc causes Fe sequestration in absorptive cells of the intestinal mucosa, as well as in macrophages, reducing Fe uptake and the ability of macrophages to release Fe resulting from erythrophagocytosis [5]. 

In the absence of pathological processes such as inflammation, infection, or neoplasia [5], the interpretation of the parameters related to the Fe status in practice will depend on physiological variations, as occurs at the reproductive level. However, the studies on Fe, Ferr, and Hepc concentrations during the estrous cycle are very limited. Indeed, although some studies in woman have shown that mean Ferr levels remain stable throughout the menstrual cycle [5,6,7,8,9,10], other investigations have not reported changes in Fe parameters [5,10] during this period. In the same way, few studies have determined changes in the activity of Hepc during the menstrual cycle [6,7], and only one study has investigated acute changes in Hepc activity after exercise in premenopausal women [11]. 

Several lines of evidence in humans have supported the relationship between high concentrations of sex steroids and Hepc production. Specifically, high estrogen concentrations have been shown to downregulate Hepc synthesis [12,13,14] and reduce Fpn expression at the cell membrane [15]. Progesterone (P4) is associated with the opposite effect [16]. Although these effects are not yet clear, it is reasonable to assume that the estrous cycle could influence the Hepc response and Fe metabolism in mares. The interpretation of Fe metabolism and Hepc measurement must depend on the stage of estrous cycle [10,11]. In equids, research on Hepc is limited, being primarily related to hypoferremia in horses treated with pentosan polysulfate [17], Freund’s adjuvant [18] and with lipopolysaccharide [19]. In these cases, hypoferremia occurs rapidly and transiently, associated with upregulated expression of hepatic Hepc mRNA, causing Fe accumulation in macrophages and reducing intestinal iron absorption without evidence of anemia or inflammation [20]. 

In women, the fluctuations of Hepc and Fe during the estrous cycle should be taken into account when these biological variables serve as judgment criteria in clinical trials and epidemiological studies for patient recruitment [8]. To date, there is no available evidence on the effect of circulating Hepc on Fe and Ferr levels in mares with physiologically normal estrous cycles. Since Hepc regulates the systemic bioavailability of Fe, and since this protein is known to be regulated by ovarian hormones in other species [21], it is important to evaluate the possible interactions between Fe, Hepc and estradiol-17β (E2), and progesterone (P4) in healthy mares in order to provide new insights into the physiology of Fe homeostasis during the estrous cycle in equids. 

## 2. Materials and Methods

### 2.1. Animals

All methods and procedures used in this study followed the guidelines of Spanish law (RD 37/2014) that regulates the protection of animals used for scientific purposes. The Animal Ethics Committee for the Care and Use of Animals of the CEU-Cardenal Herrera University (Spain) concluded that the proposed study did not need ethical approval, since this experiment was part of the clinical evaluation of the animals at this stage of their cycle (CEEA 22/01). Thirty-one cyclic non-pregnant mares, wormed and vaccinated, ranged in age from 4 to 15 years, were used in the present study. The inclusion criteria of mares were the following: (1) reproductive history: the presence of normal cyclicity during the previous breeding seasons, absence of reproductive pathologies, such endometritis, pyometra or other processes related to the loss of fertility or ability to delivery viable foals; (2) no evidence of disease within a month of the start of the study, and no treatment with antibiotics or anti-inflammatory drugs. Feeding and management practices were identical for all mares. Mares were fed 2–3 kg of concentrate twice daily, along 2–3 kg of alfa-alfa hay and wheat straw. Water was available ad libitum.

To ensure the physiological integrity of the reproductive barrier against external contaminants, transrectal and transvaginal palpation and vaginoscopic examination of the vulva, vaginal vestibule, vagina, and cervix were performed. 

Oestrus in mares was detected in the presence of the stallion and by transrectal ultrasonography of the uterus and ovaries. Mares in heat were receptive to the stallion, had uterine edema, and a follicle 35 mm in diameter or greater in the ovary. To monitor enlargement of the preovulatory follicle, the presence of uterine edema, and to predict ovulation time, transrectal ultrasonography, using a 5 MHz probe (Sonosite 180 Plus) was performed.

### 2.2. Blood Samples

Blood samples were obtained when a preovulatory follicle reached the maximum diameter (day 0) and at +5 and +16 post ovulation, as observed by ultrasound evaluation. Blood collections were always performed by jugular venipuncture, between 8:00 and 11:00 a.m., using 20 mL disposable syringes, with a Luer tip (Becton Dickinson Discardit^®^ II) attached to 40 mm 18–20 G needles (Sterican^®^, Braun Melsungen AG, Melsungen, Germany). A total 20 mL of blood was collected, and each blood sample was added to glass tubes with clot activators and polystyrene granules to collect serum (Tapval^®^). Samples were refrigerated at 4 °C for transport, then were centrifuged at 3500 rpm for 10 min (P Selecta^®^ Centrifuge), and the serum obtained was stored at −20 °C until analyzed.

### 2.3. Determination of Serum iron (Fe), Ferritin (Ferr), Hepcidin (Hepc), 17β-Estradiol (E2) and Progesterone (P4) Concentrations

Fe and Ferr (µg/dL) concentrations were analyzed by a Spin 200E spectrophotometer using commercial house reagents based on colorimetry for Fe (FerroZine) and turbidimetry for Ferr (Latex) (Spinreact^®^, Barcelona, Spain). The sample detection limits for Fe and Ferr were 0.850 µg/dL to linearity limit of 1000 µg/dL, and 5.04 µg/L, respectively. The intra- and interassay coefficients of variation (CVs) were 0.79% and 3.17%, and 5.1% and 6.3%, for Fe and Ferr, respectively.

Serum Hepc (ng/mL) concentrations were analyzed according to the manufacture’s recommendations used and equine hepcidin (HPC) enzyme-linked immunosorbent assay (ELISA) kit (MyBioSource.com, San Diego, CA, USA). The detection range was 15.6–500 ng/mL, the sensitivity was 2.0 ng/mL and the intra- and inter-assay CVs were <15%.

Plasma E2 (ng/mL) concentrations were determined by a competitive enzyme -linked immunosorbent assay (E2 Sensitive, Demeditec ELISA DE4399) specifically validated in the equine species [22]. The limit of detection was 1.4 ng/mL. The percentage of recovery in plasma was 98.72%, the standard range was paired to 25–2000 pg/mL and sensitivity analytical equal to 10.6 pg/mL. The intra- and interassay CVs at low and high concentrations were 7.87 and 5.52%. Plasma P4 (ng/mL) concentrations were determined using a solid-phase I-125 radioimmunoassay (RIA) (Coat—a -Count P4, Diagnostic Products Corporation, Los Angeles, CA, USA). The intra- and inter-assays for P4 were as follows: 16.1% and 4.3% at 3.5 nmol/L; 7.3% and 8.5% at 22.5 nmol/L; 23.3% and 6.4% at 54.8 nmol/L. The minimum detection limit was 0.1 ng/mL [23,24].

### 2.4. Statistical Analyses

Descriptive statistics including mean, maximum, and minimum values and standard deviation (SD) of all variables were obtained. The normality and homoscedasticity of the data were verified using the Kolmogorov–Smirnov and Levene tests. Logarithmic transformations of the data were performed to achieve homogeneity of variance. A one-way analysis of variance (ANOVA) was performed to compare the Fe, Ferr, and Hepc and sex hormones (E2 and P4) among day −5, 0, +5 and +16 post ovulation days, and the comparisons of means were made using the Tukey HSD test. The interrelationships between iron status markers and sex hormones were examined by linear regression analysis and the correlation was expressed by Pearson’s correlation coefficients. Statistical significance was set at *p* < 0.05. 

## 3. Results

Table 1 shows the descriptive statistics of Fe, Ferr, Hepc, E2, and P4 concentrations at day −5, day 0 and at +5 and +16 post-ovulation days, in cyclic mares. 

**Figure 1:**
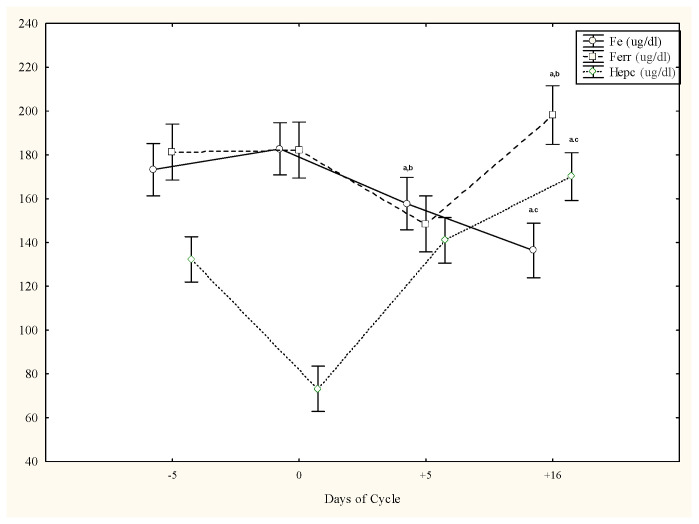
Circulating iron, ferritin, and hepcidin concentrations (mean ± SD) in cycling Spanish Purebred mares. Letters indicate significant differences among days: a vs. −5; b vs. 0; c vs. +5 (*p* < 0.05).

Compared to days −5 and 0, Fe concentrations were significantly lower on day +5 and day +16 (*p* < 0.05). Ferr and Hepc concentrations were significantly higher on day +16 than days −5 and +5 (*p* < 0.05) (Figure 1).

**Figure 2:**
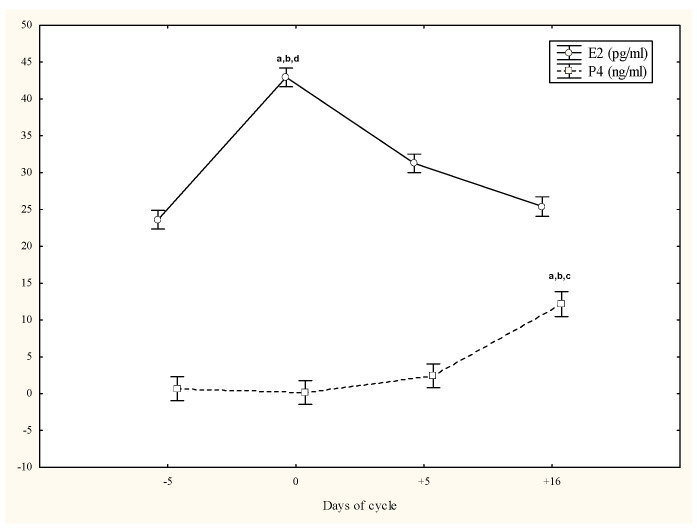
Circulating estradiol-17β (E2) and progesterone (P4) concentrations (mean ± SD) in cycling Spanish Purebred mares. Letters indicate significant differences among days: a vs. −5; b vs. 0; c vs. +5; d vs. +16 d (*p* < 0.05).

E2 concentrations were higher on day 0 than on days −5, +5 and +16 (*p* < 0.05), while P4 concentrations were higher on day +16 than on days 0 and +5 (*p* < 0.05) (Figure 2).

In Table 2, Fe and Ferr were positively correlated (r = 0.53). Fe and Ferr were negatively correlated with Hepc (r = −0.72 and r = −0.02, respectively). E2 and P4 were negatively and positively correlated with Hepc (r = −0.75 and r = 0.54, respectively).

## 4. Discussion

This study reveals associations between steroid hormones (E2 and P4) and markers of systemic Fe metabolism (Fe, Ferr, Hepc) measured at different phases of the estrus cycle (follicular and luteal phases) in cycling Purebred Spanish mares. 

The most relevant results have been the following: (1) In the follicular phase, Fe, and Ferr concentrations increase without exceeding the reference ranges, and Hepc concentrations decrease in association with the increase of E2; (2) In the luteal phase, Fe and Ferr concentrations decrease, and Hepc concentrations increase in association with the increase of P4.

### 4.1. Effects of Estrous Cycle on the Fe and Ferr Homeostasis

Compared to days +5 and +16, serum Fe and Ferr concentrations simultaneously increased in Purebred Spanish mares with the decrease of Hepc on days −5 and 0. These results for Fe and Hepc differ completely from those reported in women during this period. In fact, Fe and Hepc simultaneously evolve during the estrous cycle at a constant rate of secretion and elimination that varies with time, resulting in a stable Hepc/Ferr ratio during the cycle [8]. These results, based on the stability of Fe and Ferr, remained constant during the menstrual cycle, as reported by others [6,7,9,10,25], especially in iron-depleted individuals (serum Ferr: <12–35 μg/L). In particular physically active females with depleted Ferr reserves have lower Hepc levels during the early follicular and mid-follicular phases [11]. Therefore, diet or supplementation could represent an effective strategy to maximize iron absorption in individuals, with low Ferr levels in these phases. However, in a study among 43 Japanese college students, serum Ferr concentration was higher at the follicular phase than the luteal phase among participants that were marginally Fe deficient (n = 23), but not in participants with adequate iron status (n = 15) [26].

The mean changes in Fe parameters and Hepc along a female’s menstrual cycle may also largely depend upon her current/baseline circulating Ferr concentrations. Indeed, acute changes in Fe and Hepc parameters before and after exercise (40 min run at 75% VO2max) in three different phases of the menstrual cycle (early follicular, mid-follicular, and luteal phase) have been recently examined. Pre-exercise circulating Fe and IL-6 values were lower in the early follicular phase compared to the luteal phase [27]. Although serum Hepc increased after exercise, it does not change throughout the cycle. Additionally, neither exercise-induced changes in serum Fe levels nor IL-6 appeared to stimulate Hepc activity after exercise. The attenuated post-exercise Hepc response was probably due to low baseline Ferr levels ranged between 25.4 ng/mL during the early follicular phase, and to 29.2 ng/mL during the luteal phase (<35 μg/mL), when represents a diagnostic cut-off for stage 1 Fe deficiency [27]. As suggested by Galetti et al. [28], Hepc was probably suppressed to promote Fe uptake in response to depletion of reserves. These inconsistencies in Fe and Ferr patterns across the menstrual cycle from different studies may be due to small sample sizes, inaccurate timing of sample collection to cycle phase, and use of cross-sectional data which compared different groups of women. 

During the early follicular phase, defined by the presence of low concentrations of E2 and P4, minimum values of Hepc in women were observed [7]. During this phase, menstrual bleeding leads to active loss of Fe, therefore low Hepc levels could compensate for this loss by facilitating efficient recycling of Fe stores and absorption from the diet. This proposal could be supported by the fact that total iron-binding capacity (TIBC) peaks [29] during the early follicular phase. Other physiological mechanisms such as fluctuations in plasma volume have been proposed as a reason for Hepc reduction in this period [30,31]. In principle, this evidence should not be directly applied to the mare, since in this species, bleeding associated with the estrous cycle does not occur.

### 4.2. Effects of Estradiol-17β and Progesterone on Fe and Ferr Homeostasis

The results obtained for E2 in cycling Purebred Spanish mares partially confirm those previously obtained in this species [30,31], although only days −5, 0 (ovulation), +5 and +16 post-ovulation were considered in this studies. Although, in this study, is widely documented that E2 concentrations in the mare begin to increase between days 14 and 16 post-ovulation, which is approximately 6 to 8 days before the next ovulation and remain elevated until 1 or 2 days before ovulation, and then decrease, reaching the lowest levels during diestrus [30,31]. In the opinion of the authors, the size that the preovulatory follicle reaches, the individual character or the age of the mare, the moment of the reproductive season, as well as the method used for hormonal determination are factors that can condition changes in plasmatic concentrations of this steroid.

Experimental evidence in women suggests that estrogens lead to a greater availability of Fe due to the inhibitory effect of this hormone on Hepc synthesis [32]. The decrease of Hepc maintains the integrity of Fpn and increases the release of Fe by duodenal enterocytes (which absorb Fe), and macrophages and hepatocytes (which store Fe) [14]. 

Although the exact mechanism by which E2 influences Fe regulation is unknown, some in vitro studies in human breast [14], human ovarian cancer cell lines with epithelial-like morphology [32], human liver cells [21,32], and rodents have suggested that E2 positively regulates the genes involved in Fe metabolism (ferroportin, lactotransferrin, ceruloplasmin ferroxidase, lipocalin 2) [14,15], probably mediated likely by downregulation of Hepc activity. In ovarian epithelial cancer cells, E2-induced downregulation of Hepc gene (HAMP) expression resulting in reduced Hepc concentration [21,32]. Furthermore, in E2-treated human liver cells, Hepc levels are reduced due to E2 binding to E2-responsive elements in the HAMP gene, suppressing gene expression [32]. In addition, in women treated with high doses of estrogen as a component of in vitro fertilization programs, marked suppression of serum Hepc (~40% decrease) results [13]. In cyclic Purebred Spanish mares, the positive correlations between E2 and Fe, and negative correlations between E2 and Hepc on days −5 and 0 could indicate that as estrogens increase, Hepc inhibition could lead to increased Fe mobilization in this species too.

While most of the available study suggests an inverse correlation between E2 and Hepc, as observed here in mares, two researchers observed an increase in HAMP gene expression and Hepc synthesis in response to E2 treatment in mice [33] and humans [34]. In ovariectomized mice, E2 could induce an increase of serum and hepatic Fe through increased HAMP gene expression, according to the differential expression of E2 receptors, such as the membrane-anchored E2 receptors GPR30, and related co-regulators in different cell types [29]. On the other hand, the correlation between E2 and Fe homeostasis may be cell-type- and specie-specific context. E2 can differentially alter iron metabolism in monocytes by an IL-6-dependent manner. Exogenous treatment of human cell lines in vitro with E2, initiated a cascade of effects beginning with an increase in IL-6 synthesis, a reduction of TNF-α, HIF-1α, and ERα followed by an increase in Hepc levels [34]. However, in uterine cells, E2 may play an important role in reducing Hepc expression to support Fe turnover during E2-induced cell growth and development (mid-late follicular phase of the menstrual cycle, days 5–14). In contrast, in immune cells, mid-cycle increases in E2 may enhance the proinflammatory response in macrophages and dendrites, increasing Hepc and Fe sequestration, as part of the anti-inflammatory response [15,34]. 

In the mare, the existence of an inflammatory response related to iron metabolism based on E2 levels during estrus remains unknown. However, despite the involvement of inflammatory cells and mediators in follicular rupture, a previous study carried out by these same researchers showed that estrus and ovulation did not appear to affect the systemic inflammatory response in the Purebred Spanish mare since the levels of serum amyloid type A and C-reactive protein (CRP) did not change compared to compared to luteal phase [24]. On the contrary, CRP concentrations are reduced due to the endogenous release of E2 during the follicular phase [35], suggesting that estrogens regulate anti-inflammatory effects, reducing the expression of cytokines and molecular adhesion [36]. Despite this, in non-synchronized estrous cycles in cows, haptoglobin (Hp) was significantly higher compared to diestrus and proestrus [37]. It seems that behavioral stress during ovulation in buffaloes, as increased oxidative activity and free radical release during estrus, could be other reasons for the increase of Hp [38]. However, Fe metabolism under these inflammatory response conditions was not evaluated in any of these studies.

Additionally, during mid-cycle ovulation, a peak of testosterone levels, with an increase by ~40%, has been recorded in eumenorrheic females [39]. Rodent models show that Hepc downregulation by testosterone occurs according to testosterone-dependent upregulation of epidermal growth factor (EGF) receptors of liver [40]. Suppressive effects of both E2 and testosterone on HAMP gene expression in some cell types may induce a progressive increase in systemic Fe concentrations along the mid-late follicular phase in eumenorrheic females [31], as it could have occurred in the mare, although it cannot be confirmed.

With slight variations, the obtained P4 concentrations on days −5, 0, +5 and +16 in Purebred Spanish mares were like those previously obtained in this species. Indeed, plasma P4 fluctuations were measured between 0.1 to 11.5 ng/mL, with minimal values of 1 ng/mL during estrus. After ovulation, around the fifth to ninth day, the P4 increases constantly reaching levels between 5 and 8 ng/mL. After luteolysis that occurs on days 13 to 14 of the cycle, P4 decreases rapidly, reaching basal levels on day 16 post-ovulation, coinciding with the onset of estrus [30,31].

However, in cycling Purebred Spanish mares, these P4 levels are positively correlated with Hepc and negatively correlated with Fe. Perhaps, P4 in addition to other factors could explain the higher Hepc levels during the early and late luteal phase, such as reported during the menstrual cycle in women [17]. Hepc expression increased ~4 h after the peak in P4 concentration and peaked ~12 h after the start of P4 administration. The late rise in Hepc, following exogenous administration of P4, suggests that an intracellular second messenger or metabolite is required to reach a critical threshold before triggering downstream effects, including increased Hepc expression. In the luteal phase, low TIBC and higher Hepc levels may be indicative of stable iron utilization [7,8,29]. These changes may be due to the direct influence of P4 on Hepc activity, or after the rebound in systemic Fe levels around ovulation to regulate iron homeostasis and prevent Fe excess [41,42]. According to Matta et al. [42], cellular and human clinical studies of hepatocytes and among women receiving P4 during in vitro fertilization, reported a positive effect of P4 on Hepc biosynthesis through binding to the membrane bound P4 receptor [17]. Consequently, the increase of Hepc during elevations of P4 levels could reveal a more conservative iron state since the Fe and Ferr levels are reduced with respect to the pre- and ovulatory periods. 

In addition, P4 exerts a natriuretic effect due to its aldosterone antagonism, resulting in plasma sodium and water loss [43,44]. Thus, hormone-associated changes in plasma volume and plasma protein concentration, that occur during the menstrual cycle, offer a possible mechanism to explain differences in iron status indicators at different phases of the estrous cycle. 

On the other hand, serum Ferr is not necessarily indicative of Fe overload as Fe and Ferr can be regulated independently of one another [41]. Instead, serum Ferr is a known marker of inflammation and cellular damage and may in fact not be ‘carrying’ much Fe at all. Hence, in horses, inflammatory cytokines such as IL-1β, IL-6, and TNFα released in response to inflammation [20] and exercise [45] lead to the secretion of Hepc, negatively influencing the transport and absorption of Fe. Additionally, Fe is a very sensitive marker of inflammation in equines; it shows rapid kinetics, decreasing a few hours after an inflammatory insult and recovering to initial values 1 to 2 days after the decrease in inflammation [46,47]. Hemolysis and/or volume expansion have been suggested as possible causes of decreased serum Fe in horses undergoing a 3-week training program [48]. In the absence of inflammation and exercise, it may be that the changes in iron status parameters could be induced by the estrous cycle in mares.

In the mare, E2-mediated inhibition of Hepc synthesis during the preovulatory and ovulatory periods allows for greater mobilization, and probably greater Fe absorption from the diet. However, during the luteal period these effects are reversed. The high concentrations of P4 in this period may have a stimulating effect on Hepc and, consequently, levels of Fe are reduced and Ferr are increased. According to these results, in cycling Purebred Spanish mares, there is a relationship between steroid hormones and the systemic metabolism of Fe. Nevertheless, additional studies will be necessary to further investigate the physiological role of these hormones in the regulation of Hepc biosynthesis at the molecular level and, consequently, in Fe metabolism in the cyclic mare.

## 5. Conclusions

In cycling Purebred Spanish mares there is clearly a relationship between steroid hormones concentrations and markers of systemic Fe metabolism. Estrogen dominance in the pre- and ovulatory periods allows for a more effective iron status mediated by hepcidin inhibition. However, the stimulatory effect of progesterone on hepcidin synthesis substantially reduces circulating levels of iron and increases its reserves. Future research is required to clarify these interrelationships at the molecular level.

## Figures and Tables

**Table 1:** Mean ± SD of serum iron (Fe), ferritin (Ferr), hepcidin (Hepc), 17-β estradiol (E2) and progesterone (P4) in cyclic Purebred Spanish mares.

Parameters	Days of Cycle	Mean ± SD	Range
Fe (μg/dL)	−5	173.2 ± 24.9	140–202
	0	182.7 ± 11.3	165–201
	+5	157.7 ± 21.2	183–188
	+16	136.6 ± 17.9	110–160
Ferr (μg/dL)	−5	181.2 ± 19.8	134–202
	0	182.2 ± 14.0	158–202
	+5	148.4 ± 32.9	56–189
	+16	198.2 ± 6.4	189–208
Hepc (μg/dL)	−5	132.2 ± 24.3	100–189
	0	73.2 ± 9.48	56–89
	+5	141.0 ± 12.92	110–159
	+16	170.1 ± 17.5	135–190
E_2_ (pg/mL)	−5	24.3 ± 0.77	22.2–25.3
	0	44.62 ± 3.58	36.9–49.0
	+5	31.3 ± 0.10	31.2–31.4
	+16	26.8 ± 1.32	23.3–27.8
P_4_ (ng/mL)	−5	0.71 ± 0.04	0.63–0.74
	0	0.17 ± 0.01	0.13–0.18
	+5	2.52 ± 0.08	2.34–2.56
	+16	7.04 ± 6.56	1.10–22.5

**Table 2:** Spearman’s coefficients among iron, ferritin, hepcidin, estradiol-17β (E2), and progesterone (P4) in cycling Purebred Spanish mares.

	Ferr (μg/dL)	Hepc (ng/dL)	E_2_ (pg/mL)	P_4_ (ng/mL)
Fe (μg/dL)	0.53	−0.72	0.40	−0.52
Ferr (μg/dL)		−0.02	−0.08	0.28
Hepc (ng/mL)			−0.75	0.54
E2 (pg/mL)				−0.36

Fe: iron; Ferr: ferritin; Hepc: hepcidin; E_2_: estradiol-17β and P_4_: progesterone.

## Data Availability

The data that support this study will be shared upon reasonable request to the corresponding author.
